# Early surgery determines recovery of motor deficits in lumbar disc herniations—a prospective single-center study

**DOI:** 10.1007/s00701-020-04614-0

**Published:** 2020-11-04

**Authors:** Nikolaus Kögl, Konstantin Brawanski, Pierre-Pascal Girod, Ondra Petr, Claudius Thomé

**Affiliations:** Department of Neurosurgery, Medical University of Innsbruck, Tyrol, Austria

**Keywords:** Paresis, Motor deficit, Recovery, Surgical timing, Disc herniation, Discectomy

## Abstract

**Background:**

Patients with intervertebral disc herniation undergo surgical removal of herniated disc material in cases of persisting symptoms and/or neurologic deficits. While motor deficits often prompt surgery, little is known about the optimal timing of surgery in these cases. The aim of this study was to prospectively evaluate the impact of timing of disc surgery on motor recovery. Does postponing surgical treatment worsen outcome?

**Method:**

In total, 120 patients with sciatica and/or sensorimotor deficits due to a lumbar disc herniation were surgically treated at the authors’ center within a 3-month period. In 60 patients, motor deficits were present at the time of admission. Motor function was assessed using manual muscle testing and subdivided according to the Medical Research Council (MRC) scale. Patient demographics, neurologic deficits, duration of motor deficits, treatment characteristics, and outcome were assessed. At a minimum follow-up of 1 year, functional recovery and complications were collated. Patients were subdivided into groups according to the severity of the paresis (MRC ≤ 3/5 vs. MRC 4/5). Intra-group differences were compared based on the duration of the neurologic deficits.

**Results:**

Patients with moderate and severe paresis (MRC ≤ 3/5) benefit from treatment within 72 h as they were shown to have a significantly higher complete recovery rate at 1-year follow-up (75% vs. 0%; *p* < 0.001).

**Conclusion:**

Immediate surgery should be offered to patients with moderate and severe motor deficits to increase the likelihood of neurologic recovery. This prospective data may have an impact on emergency triage in these patients.

## Introduction

The most common cause of sciatica is intervertebral disc herniation or spondylosis leading to mechanical nerve affection [6, 15]. The annual incidence in the general population that experience sciatica ranges from 1 to 5% [9, 14]. The initial treatment of sciatica is conservative due to its favorable natural history as symptoms usually decrease or disappear in 60–80% within 3 months[3, 26]. In approximately 20% of cases, patients undergo surgical removal of herniated disc material because of persisting symptoms, which is usually done within 6–26 weeks in western countries as surgery is economically affordable and associated with only minor risks [3]. Improvement in leg pain is achieved much faster for patients in case of early surgery [22]. Nevertheless, short-term benefits of early surgery level out in medium to long-term follow-up compared to conservatively treated patients [11, 21–23].

Neurological deficits such as paresis can be seen in up to 30 to 50% of patients with symptomatic disc herniation [25]. The recovery rate varies widely in the literature with little information on optimal timing as mild motor deficits may remain stable or even recover with conservative management [25]. Cauda equina syndrome represents a surgical emergency and should be treated within 48 h as postponing treatment may result in rectal and urinary incontinence. Only 40% of patients treated after this timeline regained the lost bladder function [1, 4].

A recently published retrospective study has also indicated superior outcome for early surgery (< 48 h) in patients with moderate and/or severe deficits according to the Medical Research Council (MRC) ≤ 3/5 [20]. The aim of this single-center study was therefore to prospectively evaluate the impact of timing of disc surgery on motor recovery.

## Methods

A multicenter registry study designed to evaluate the need for an annular closure device (ACD) prospectively assessed lumbar disc herniation patients. The authors enriched the respective single-center data set to analyze patients with motor deficits regarding severity and timing of surgery. After obtaining approval by the institutional ethics committee, this study enrolled consecutive patients from September 2015 to December 2015 with a follow-up of at least 1 year. In total, 120 patients with persistent pain and/or neurologic deficit were treated by lumbar microscopic sequestrectomy/discectomy within this period of time. Detailed information including demographics, medical history, surgery details, radiographic data, and neurologic examination were collected and analyzed. The duration of motor deficits and the existence of a cauda equina syndrome were precisely documented as clearly stated by the patients. In case of unknown duration of motor deficits, the patient was not enrolled in this study. Preliminary prospective data of our group had indicated no complete recovery of severe deficits after 72 h, so that this cut-off value was chosen for analysis. In accordance with clinical guidelines, patients were subdivided into groups according to the severity of the paresis (MRC ≤ 3/5 vs. MRC 4/5) [20]. A mild motor deficit was defined as MRC grade 4. This is characterized as active movement against gravity and resistance during manual testing. Grade 3 was defined as moderate, whereas grades 0–2 were recorded as a severe deficit. The degree of sensory and motor impairment was preoperatively assessed using standard methods. To overcome the challenge of distinguishing between pain-induced inhibition of motor function and true motor deficit, analgesics were prescribed and the patients’ symptoms were reevaluated. The clinical information was obtained by manual testing as this is not inferior to those documented by EMG [24]. Surgery was performed by several surgeons at the authors’ center.

At a minimum follow-up of 1-year functional recovery, residual sciatica and complications were assessed. Follow-up visits were scheduled at 6 weeks and 1 year after surgery. Neurologic examination and manual testing were performed by a single surgeon to minimize interobserver variability.

Complications were recorded as intraoperative, postoperative, and overall complications.

### Exclusion criteria

We excluded all patients, who did not sign the informed consent form (ICF). Underage patients (< 18 years of age) and procedures in the thoracic and cervical spine were also not included in this analysis.

### Perioperative data

The duration of neurologic deficits, the quality of the sensory deficit, and the existence of cauda equina syndrome were evaluated before and after surgery. Any changes were documented over time. Surgical details such as type of discectomy, intraoperative findings, the use of an annular closure device, and prior surgeries were also documented. All intraoperative and perioperative complications were noted. In case of complications, all additional treatments including the need and time point of revision surgery were documented.

Clinical outcomes including neurologic impairment were evaluated statistically.

## Statistics

Endpoints were analyzed as appropriate in dependence on the data distribution at a two-sided 0.05 level of significance. Detailed descriptive statistics were provided for the data collected and 95% confidence intervals were calculated for all relevant estimates. Measurements concerning the time course of follow-up were analyzed by ANCOVA or generalized model alternatives for categorical or semi-quantitative data. Distributions of numeric variables were given as mean and (±) standard deviation. The Mann-Whitney *U* test was used to asses intergroup differences of continuous measures. Chi-square or Fisher exact tests were used for dichotomous data analysis depending on the number of subjects involved. A *p* value of less than 0.05 was considered statistically significant. Data is analyzed for normality by the Kolmogorov-Smirnov test. The Wilcoxon signed-rank test was used for paired data and for the analysis of intragroup changes.

All data was pseudonymized as soon as clinically reasonable. Data entry in an electronic database (SPSS Statistics 25; IBM, Armonk, New York, USA) was performed with pseudonymized data stored according to local regulations in a database.

## Results

One hundred and twenty microscopic disc surgeries were performed within 3 months and 119 (99.2%) were followed up for at least 1 year. The most common level of disc herniation was L4/5 (56 patients; 46.7%).

### Demographics

Sixty-four patients were female (53.3%) and the mean age was 49.6 ± 15.6 years.

Twenty-five patients (20.8%) were previously treated with at least one prior surgery at the index level (Table 1). Persistent pain and acute exacerbation of the symptoms led to surgery in 48.3% (58) of the cases. Fifty percent of all patients (60) presented with a motor deficit. Of those, thirty-five patients (58.3%) presented with a mild motor deficit (MRC 4/5), whereas 25 patients (41.7%) suffered from a moderate or severe (≤ 3/5) paresis. Twelve patients (10.0%) suffered from an additional adjacent myotome paresis caused by the simultaneous affection of the traversing and exiting nerve roots. Sensory deficits were documented in 94 cases (78.3%). A cauda equina syndrome was diagnosed in six cases (5.0%) (Table 2).

**Table 1:** Demographics and patients’ characteristics

Gender	*N*
Female	64 (53.3%)
Male	56 (46.7%)
	Mean
Age (years)	49.6 ± 15.9 (19–84)
BMI	26.0 ± 4.0 (18.3–37.4)
Index Level	*N*
L2/3	3 (2.5%)
L3/4	8 (6.7%)
L4/5	56 (46.7%)
L5/6	1 (0.8%)
L5/S1	46 (38.3%)
L1/2 + L2/3	1 (0.8%)
L3/4 + L4/L5	2 (1.7%)
L3/4 + L5/S1	1 (0.8%)
L4/5 + L5/S1	2 (1.7%)
Prior surgery at index level	*N*
Primary herniation	95 (79.2%)
Recurrence	16 (13.3%)
Second reherniation	8 (6.7%)
Multiple surgeries at index level	1 (0.8%)
Total	120 (100%)

**Table 2:** Sensorimotor deficits

Motor deficit	*N*
No motor deficit	60 (50.0%)
MRC > 3/5 paresis	35 (29.2%)
MRC ≤ 3/5 motor deficit	25 (20.8%)
Sensory deficit	*N*
No sensory deficit	26 (21.7%)
Paraesthesia	10 (8.3%)
Hypesthesia	82 (68.3%)
Dysesthesia	2 (1.7%)
Cauda equina syndrome	*N*
No vesicorectal disorder	114 (95.0%)
Vesicorectal disorder	6 (5.0%)

### Outcome

**Fig. 1:**
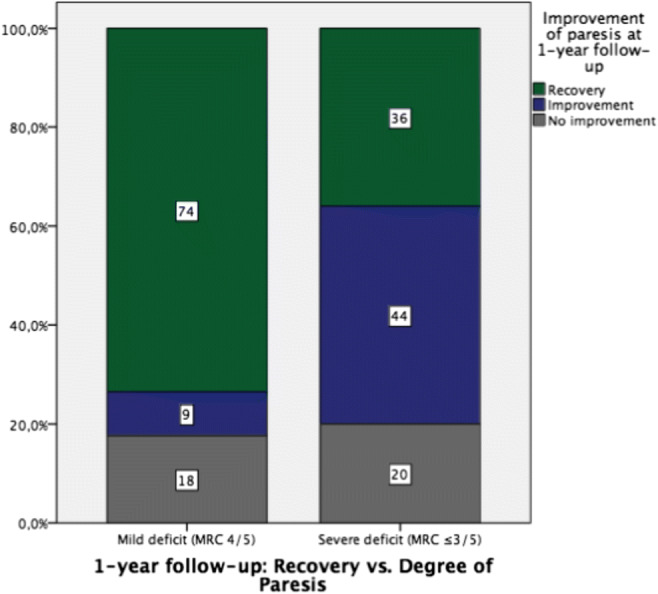
Difference of recovery of mild vs. moderate and/or severe motor deficits irrespective of surgical timing

**Fig. 2:**
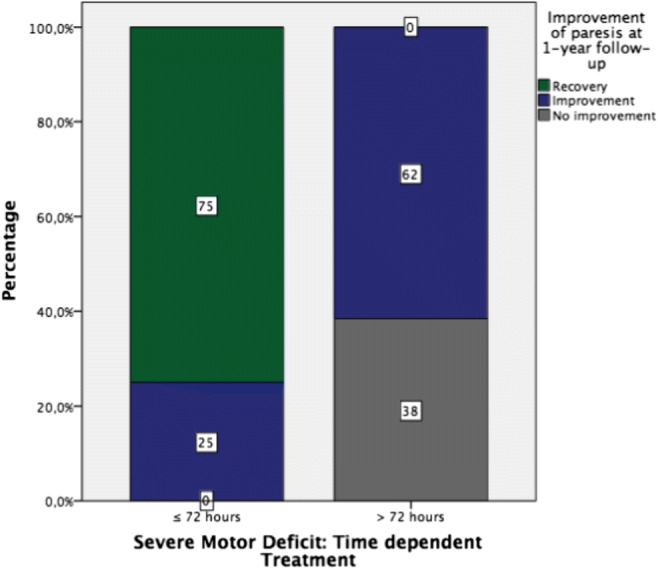
Recovery of moderate and/or severe motor deficits (MRC ≤ 3/5) depending on surgical timing

Recovery of the initial motor deficit was seen in 25 patients (41.7%) at regular follow-up after 6 weeks. At 1-year follow-up examination, 35 (58.3%) of all patients with initial motor deficit regained full strength (Fig. 1). Twenty-five (41.7%) patients initially presented with a moderate and/or severe motor deficit (≤ 3/5 MRC). Thirteen of those (52.2%) were treated later than 72 h after paresis onset. Notably, none of them (0.0%) completely regained full strength of the affected myotome (Fig. 2). In contrast to this subgroup, 9 out of 12 patients (75%) treated within 72 h recovered completely (*p* < 0.001) (Table 3). In case of moderate and/or severe motor deficits, the mean duration of paresis prior to surgery varied significantly between patients, who recovered and those who did not regain full strength (mean 1.9 ± 0.8 vs. 15.2 ± 23.8 days). Furthermore, none of the patients with an initial deficit grade MRC 0 or 1 recovered completely—independent on surgical timing.

**Table 3:** Time-dependent recovery of LDH associated motor deficits

Duration of ≤ 3/5 (MRC) motor deficit	Incomplete recovery (*n*)	Complete recovery after 1a (*n*)	Total	p*
Paresis ≤ 72 h	3	9	12	
Paresis > 72 h	13	0	13	
Total	16	9	25	< 0.001
Duration of 4/5 (MRC) motor deficit	Incomplete recovery (*n*)	Complete Recovery after 1a (*n*)	Total	p*
Paresis ≤ 72 h	0	9	9	
Paresis > 72 h	9	16	25	
Total	9	25	34	0.073

**Fig. 3:**
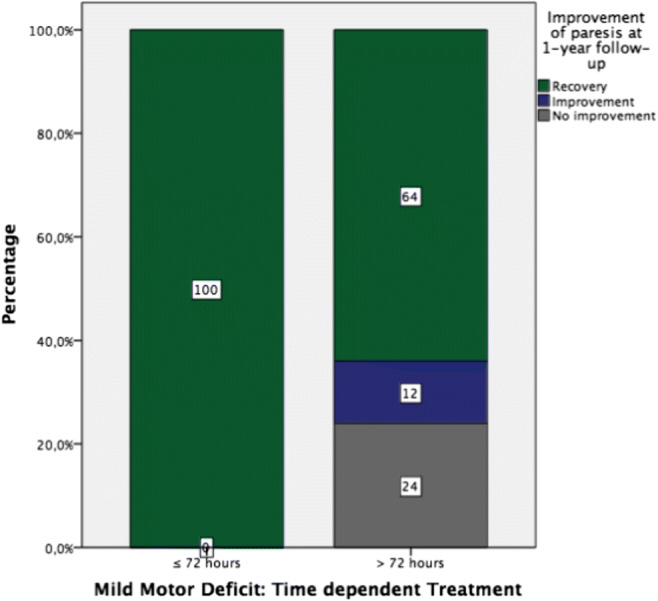
Recovery of mild motor deficits (MRC 4/5) depending on surgical timing

All patients (9) with a mild motor deficit (MRC 4/5), who were treated surgically within 72 h after onset, recovered. However, only 64% of these patients treated after this time window regained full strength at 1-year follow-up (*p* = 0.073) (Fig. 3).

Analysis of sensory deficits did not reveal a significant impact of surgical timing.

Six patients (5%) presented with a cauda equina syndrome with symptomatic vesicorectal dysfunction. Four patients (3.3%) had experienced these symptoms for less than 24 h before admission and emergency discectomy. None of them showed any sequelae at 1-year follow-up. Unfortunately, two patients, who presented with vesicorectal dysfunction preexisting for more than 2 days and even a week, did not recover completely after decompression (*p* = 0.05).

Ninety-four patients (78.3%) initially presented with any kind of sensory deficit. Forty-three patients (44.8%) recovered from their sensory deficit 6 weeks after surgery, whereas 58 patients (60.4%) recovered their sensory deficits after 1 year.

### Complications

Complications occurred in 24 patients (20%). The most common complication was a symptomatic reherniation, which was diagnosed based on clinical and radiologic findings in 14 cases (11.7%). Of these, ten patients (8.3%) underwent revision surgery within 1 year. The other four patients were successfully treated conservatively with complete recovery. An accidental durotomy occurred in 5 patients (4.2%), none of them requiring revision surgery. Furthermore, two patients (1.7%) did undergo fusion surgery for L5/S1 instability within 6 months after sequestrectomy. There was one revision for epidural hematoma and two patients (1.7%) required drainage of a seroma.

## Discussion

Our prospective study of 120 patients evaluating the impact of very early treatment, defined as surgical decompression of the affected nerve root within 72 h after symptom onset, showed significantly better outcome at 1-year follow-up in patients treated in an emergency manner. Patients suffering from a moderate to severe motor deficits (MRC ≤ 3) highly benefit from very early discectomy as no one treated 72 h after onset regained full strength, whereas 75% decompressed within 72 h recovered completely. Also, there was a clear trend towards better outcome in patients with a mild motor deficit, if surgery was performed within 72 h after symptom onset. This is highly relevant in case of a symptomatic quadriceps palsy in order to prevent falls and further injuries, especially in active patients or people working in heights. However, these findings are controversial to previous publications including the official recommendations by the German Society of Neurology (DGN), which indicates surgery in case of moderate or severe paresis [10]. Petr et al. [20] defined surgery within 48 h as early intervention, based on the recovery rate of cauda equina [20]. However, preliminary data of this study revealed incomplete recovery of moderate and/or severe paresis, if surgery was performed after a 72-h lapse of time.

In our study, 50 % of all patients presented with a lumbar disc herniation and accompanying motor deficit. This distribution within the study population is in line with literature as motor function impairment due to a LDH is found in 40–82% [19]. Of note, the series was initially enrolled to determine the need for an ACD; therefore, no conservatively treated control group exists. Due to intraoperative findings and implantation criteria, none of the patients with motor deficits received an ACD.

The majority of previous studies focused on whether to surgically treat persistent sciatica, but the role and timing of surgical intervention in case of motor deficits remained unclear. Superiority in short-term recovery of sciatica was shown for the surgical group [5, 28]. Yet, this early positive effect leveled out to a non-significant difference in the long term [5, 18, 21, 24].

Aono et al. [2] retrospectively demonstrated better outcome in early treated patients with a high degree paresis of ankle dorsiflexion. However, the earliest time point of surgery was 4 days after onset and only 19% reached an excellent result by regaining full strength [2]. An inverse relation between the degree of recovery of motor function and preoperative severity as well as symptom duration was described by Postacchini et al. [24]. The authors described an 84% recovery rate in patients with a mild and 61% rate in those with a severe motor deficit. No recovery was seen in patients undergoing surgery later than 3 months [24]. Lønne et al. [16] reported the degree of preoperative paresis as a significant risk factor as a severe paresis quadruplicated the risk of incomplete recovery. Timing was of peripheral importance, but surgery was performed late [16]. Dubourg et al. [7] compared the recovery rate between medically and surgically treated patients with a severe (≤ 3/5 MRC) paresis. However, a longer course of sciatica, different types of herniation, and higher number of paretic muscles in the surgically treated group might have substantially limited the results. The authors reported no significant benefit in patients treated surgically with a motor deficit lasting for less than a month [7].

Recently, our group reported substantially better outcome in patients with severe motor deficits in a retrospective analysis if surgically treated within 48 h [20].

In this study, none of the affected muscles recovered completely, if nerve decompression was achieved after 72 h in patients with a severe paresis (MRC ≤ 2/5), whereas all patients with mild motor deficits treated within 72 h made full motor function recovery.

Importantly, we identified the degree and duration of motor deficits to be a paramount predictor of residual deficits. While our findings regarding the sensory deficits are in line with the other recent studies [13, 27], the predictors for recovery of sensory deficits remain unclear. Notably, the sensory impairment does not seem to affect quality of life as much as persistent pain [13, 27].

In order to distinguish between a pain-induced erratic motor response and a neurologic motor deficit, analgesics were prescribed prior to reevaluation. In some cases, the patients’ position and way of testing required modification.

The need for emergency surgical treatment has been reported in case of cauda equina syndrome [1, 4]. In our study, recovery of bladder and bowel dysfunction was achieved only in patients treated within the first 24 h after onset, which is in line with abovementioned studies [1, 4].

In summary, we are able to prospectively demonstrate the importance of early surgical intervention. This particular information can predict the likelihood of recovery and therefore should be used for patient management or triage.

The right indication and timing for surgery is pivotal to prevent persistent neurologic deficits and pain, as they potentially cause disability, substantially affecting the time to return to work and quality of life. Albeit short-term increased medical costs due to surgical treatment, operation, and hospital stay, surgical treatment is associated with lower overall costs [12].

## Limitations

The surgical timing in case of motor deficits remains controversial, being affected by several factors. Of note, the exact time of onset is difficult to assess unless it significantly influences the patient’s daily life and or mobility. Furthermore, the evaluation and degree of motor deficit varies across different centers, impeding comparison to other studies [19]. A limitation is the MRC scale as it is non-continuous. A grade of “4,” for example, encompasses a very large span of muscle weakness, ranging from minimal weakness to significant disability. This can be caused by pain provocation, poor compliance, or subjective clinical judgment [8, 17]. This prospective study is missing a conservative control group, as it focused on the time-dependent outcome of LDH-associated sensorimotor deficits. In spite of a small number, this study clearly indicates superior motor function recovery, if surgery is being performed within a 72-h period.

## Conclusions

Early surgical treatment must be advised in case of severe motor deficits and/or cauda equina syndrome to allow complete and fast recovery. We identified the degree and duration of motor deficits to be a paramount predictor of residual deficits. In particular, patients with short-lived moderate and severe motor deficits should undergo surgery within 72 h after onset. Even in patients with a mild weakness, early surgical intervention showed a clear trend towards better outcome, which can be highly relevant depending on the disability resulting from the deficit.
